# Supplementary material for: Characterizing subgroups of sexual behaviors among men who have sex with men eligible for, but not using, PrEP in the Netherlands
Source: PLoS One. 2023 Apr 6;18(4):e0284056. doi: 10.1371/journal.pone.0284056 (PMC10079044; doi:10.1371/journal.pone.0284056)
Supplement: S1 Table — Abbreviations: AIC = Akaike’s information criterion; BIC = Bayesian information criterion *Entropy could not be calculated. (DOCX) [file pone.0284056.s001.docx]

**S1 Table. Comparison of fit statistics for Latent Class Analysis with 1-6 classes.**

| **Number of classes** | **AIC** | **BIC** | **Entropy value** |
| --- | --- | --- | --- |
| 1 | 174591.3 | 174626.2 | 1.00 |
| 2 | 164807.4 | 164886.0 | 0.55 |
| 3 | 164539.2 | 164661.4 | 0.79 |
| 4 | 164506.3 | 164637.2 | 0.02 |
| 5 | 164490.5 | 164630.2 | * |
| 6 | 164483.5 | 164649.3 | * |

Abbreviations: AIC=Akaike's information criterion; BIC=Bayesian information criterion

*Entropy could not be calculated.
