# Supplementary material for: Characterizing subgroups of sexual behaviors among men who have sex with men eligible for, but not using, PrEP in the Netherlands
Source: PLoS One. 2023 Apr 6;18(4):e0284056. doi: 10.1371/journal.pone.0284056 (PMC10079044; doi:10.1371/journal.pone.0284056)
Supplement: S2 Table — Parameter estimates for the associations between STI diagnosis or sociodemographic variables and latent classes were directly obtained from a generalized structural equation model. Explanation of data: OR = odds ratio; aOR = adjusted odds ratio; 95% CI = 95% confidence interval. *aOR: all models were adjusted for the variables present in the table. **Any STI includes anal chlamydia, anal gonorrhea, hepatitis C virus, hepatitis B virus, and syphilis diagnosed at the visit. ***Originating from an STI/HIV endemic area is defined as being born in and having either one or both parents born in Suriname, Turkey, Netherlands Antilles, North Africa, Sub-Saharan Africa, Eastern Europe, Central and South America, or Asia. ****Region is defined as urban, referring to all “Randstad” provinces, or non-urban, referring to all other provinces. (DOCX) [file pone.0284056.s002.docx]

**S2 Table. Association between class membership and various factors (LCA model with covariates) comparing class 2 and 3.**

|  | **Class 3**  *Vs. class 2* | | | |
| --- | --- | --- | --- | --- |
|  | *OR* | *95% CI* | *aOR** | *95%CI* |
| **Any STI****  *Yes vs. no* | 1.8 | 1.5-2.1 | 1.3 | 1.2-1.5 |
| **Age**  *≥36 vs. ≤35 years* | 2.6 | 2.1-3.2 | 1.8 | 1.6-2.0 |
| **Sexual partner(s)**  *Male and female vs. male* | 1.8 | 1.3-2.3 | 0.4 | 0.4-0.5 |
| **Region*****  *Urban vs. non-urban* | 1.0 | 0.9-1.1 | 0.7 | 0.6-0.8 |
| **From an STI/HIV endemic area******  *Yes vs. no* | 0.8 | 0.8-0.9 | 1.3 | 1.1-1.5 |
| **Education level**  *High vs. low-middle* | 0.7 | 0.6-0.8 | 0.9 | 0.8-1.1 |

Parameter estimates for the associations between STI diagnosis or sociodemographic variables and latent classes were directly obtained from a generalized structural equation model. Explanation of data: OR = odds ratio; aOR = adjusted odds ratio; 95% CI = 95% confidence interval. *aOR: all models were adjusted for the variables present in the table. **Any STI includes anal chlamydia, anal gonorrhea, hepatitis C virus, hepatitis B virus, and syphilis diagnosed at the visit. ***Originating from an STI/HIV endemic area is defined as being born in and having either one or both parents born in Suriname, Turkey, Netherlands Antilles, North Africa, Sub-Saharan Africa, Eastern Europe, Central and South America, or Asia. ****Region is defined as urban, referring to all “Randstad” provinces, or non-urban, referring to all other provinces.
