# Supplementary material for: Characterizing subgroups of sexual behaviors among men who have sex with men eligible for, but not using, PrEP in the Netherlands
Source: PLoS One. 2023 Apr 6;18(4):e0284056. doi: 10.1371/journal.pone.0284056 (PMC10079044; doi:10.1371/journal.pone.0284056)
Supplement: S3 Table — Explanation of data: Models were estimated using maximum likelihood, which was calculated by summing all conditional likelihoods of each latent class multiplied by the associated latent class probabilities. The posteriori probability of a visit i belonging to each class k, πik, was determined from this likelihood. Visits were then assigned a latent class k corresponding to the highest probability πik. Data are presented as percentages (n). (DOCX) [file pone.0284056.s003.docx]

**S3 Table. Class membership size (number of visits) across three latent classes, stratified by year (2019, 2020 and 2021).**

|  | **Class 1** | **Class 2** | **Class 3** |
| --- | --- | --- | --- |
| **2019** | 50.4% (n=7,424) | 34.1% (n=5,025) | 15.5% (n=2,280) |
| **2020** | 53.0% (n=10,715) | 29.4% (n=5,944) | 17.6% (n=3,547) |
| **2021** | 58.4% (n=6,216) | 24.8% (n=2,640) | 16.8% (n=1,791) |
| **2019-2021 (all years)** | 53.5% (n=24,383) | 29.8% (n=13,596) | 16.7% (n=7,603) |

Explanation of data: Models were estimated using maximum likelihood, which was calculated by summing all conditional likelihoods of each latent class multiplied by the associated latent class probabilities. The posteriori probability of a visit *i* belonging to each class *k*, *π_ik_*, was determined from this likelihood. Visits were then assigned a latent class *k* corresponding to the highest probability *π_ik_*. Data are presented as percentages (n).
