# Supplementary material for: Characterizing subgroups of sexual behaviors among men who have sex with men eligible for, but not using, PrEP in the Netherlands
Source: PLoS One. 2023 Apr 6;18(4):e0284056. doi: 10.1371/journal.pone.0284056 (PMC10079044; doi:10.1371/journal.pone.0284056)
Supplement: S4 Table — Parameter estimates for the associations between STI diagnosis or sociodemographic variables and latent classes were directly obtained from a generalized structural equation model. Explanation of data: OR = odds ratio; aOR = adjusted odds ratio; 95% CI = 95% confidence interval. *aOR: all models were adjusted for the variables present in the table. **Any STI includes anal chlamydia, anal gonorrhea, hepatitis C virus, hepatitis B virus, and syphilis diagnosed at the visit. ***Originating from an STI/HIV endemic area is defined as being born in and having either one or both parents born in Surinam, Turkey, Netherlands Antilles, North Africa, Sub-Saharan Africa, Eastern Europe, Central and South America, or Asia. ****Region is defined as urban, referring to all “Randstad” provinces, or non-urban, referring to all other provinces. (DOCX) [file pone.0284056.s004.docx]

**S4 Table. Association between class membership and various factors (LCA model with covariates) comparing class 2 and 3 to class 1; using one randomly selected visit per individual.**

|  | **Class 2**  *Vs. class 1* | | | | **Class 3**  *Vs. class 1* | | | |
| --- | --- | --- | --- | --- | --- | --- | --- | --- |
|  | *OR* | *95%CI* | *aOR** | *95%CI* | *OR* | *95% CI* | *aOR** | *95%CI* |
| **Any STI****  *Yes vs. no* | 1.1 | 1.0-1.3 | 1.5 | 1.2-1.9 | 1.9 | 1.6-2.2 | 1.8 | 1.6-2.09 |
| **Age**  *≥36 vs. ≤35 years* | 1.3 | 1.2-1.6 | 1.2 | 1.0-1.4 | 2.2 | 2.0-2.4 | 2.2 | 2.0-2.5 |
| **Sexual partner(s)**  *Male and female vs. male* | 3.4 | 2.8-4.1 | 4.0 | 3.2-5.1 | 0.5 | 0.4-0.8 | 1.7 | 1.4-2.1 |
| **Region*****  *Urban vs. non-urban* | 1.5 | 1.3-1.74 | 2.6 | 2.1-3.2 | 1.4 | 1.3-1.6 | 1.8 | 1.6-2.1 |
| **From an STI/HIV endemic area******  *Yes vs. no* | 1.0 | 0.9-1.1 | 0.6 | 0.5-0.8 | 0.8 | 0.73-0.9 | 0.7 | 0.7-1.2 |
| **Education level**  *High vs. low-middle* | 1.4 | 1.0-1.8 | 1.0 | 0.8-1.2 | 0.9 | 0.7-1.2 | 0.9 | 0.8-1.0 |

Parameter estimates for the associations between STI diagnosis or sociodemographic variables and latent classes were directly obtained from a generalized structural equation model. Explanation of data: OR = odds ratio; aOR = adjusted odds ratio; 95% CI = 95% confidence interval. *aOR: all models were adjusted for the variables present in the table. **Any STI includes anal chlamydia, anal gonorrhea, hepatitis C virus, hepatitis B virus, and syphilis diagnosed at the visit. ***Originating from an STI/HIV endemic area is defined as being born in and having either one or both parents born in Surinam, Turkey, Netherlands Antilles, North Africa, Sub-Saharan Africa, Eastern Europe, Central and South America, or Asia. ****Region is defined as urban, referring to all “Randstad” provinces, or non-urban, referring to all other provinces.
