# Supplementary material for: Characterizing subgroups of sexual behaviors among men who have sex with men eligible for, but not using, PrEP in the Netherlands
Source: PLoS One. 2023 Apr 6;18(4):e0284056. doi: 10.1371/journal.pone.0284056 (PMC10079044; doi:10.1371/journal.pone.0284056)
Supplement: S1 Fig — Explanation of data: models were estimated using maximum likelihood, which was calculated by summing all conditional likelihoods of each latent class multiplied by the associated latent class probabilities. The posteriori probability of a visit i belonging to each class k, πik, was determined from this likelihood. Visits were then assigned a latent class k corresponding to the highest probability πik. The figures show the distribution of probabilities for belonging to a class given the assigned class membership. Each point represents an individual consultation visit. Figure A shows the probabilities for those assigned to class 1. Figure B shows the probabilities for those assigned to class 2. Figure C shows the probabilities for those assigned to class 3. For example, the dots in the figure indicate that only few visits had a lower probability of belonging to a given class, and the longer lines (subsequent dots), indicate that the majority of visits had (almost) 100% probability of belonging to a given class, and a very low probability of belonging to another latent class. (DOCX) [file pone.0284056.s005.docx]

**S1 Fig. The a posteriori probabilities of class membership per class.**

| **A** | **B** |
| --- | --- |
| 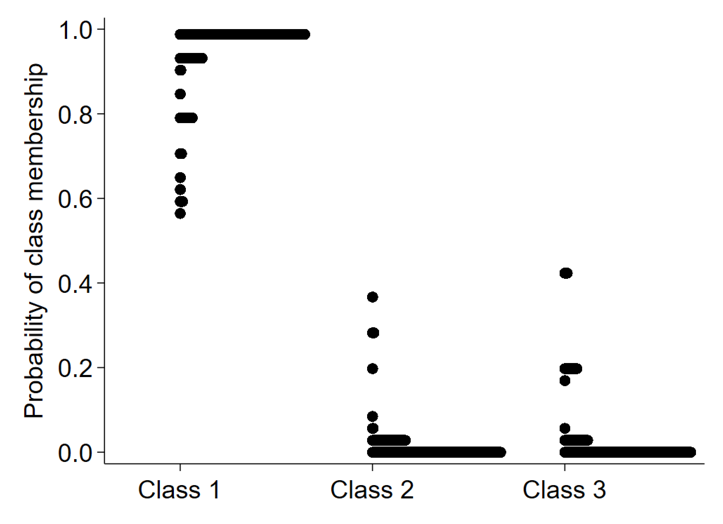 | 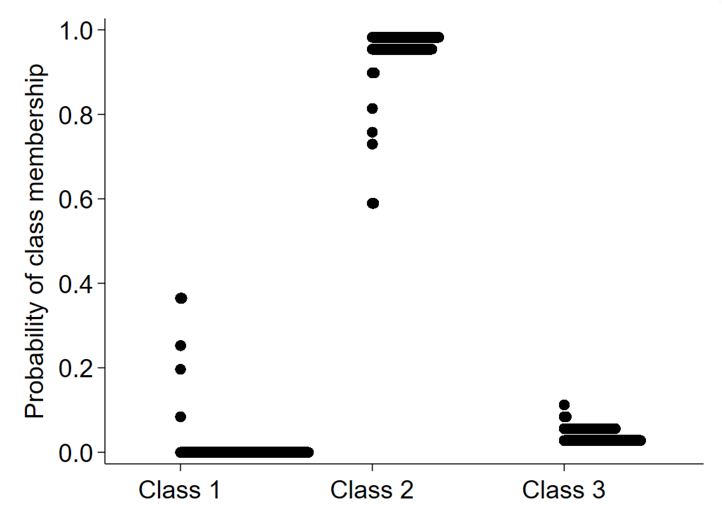 |
| **C** |  |
| 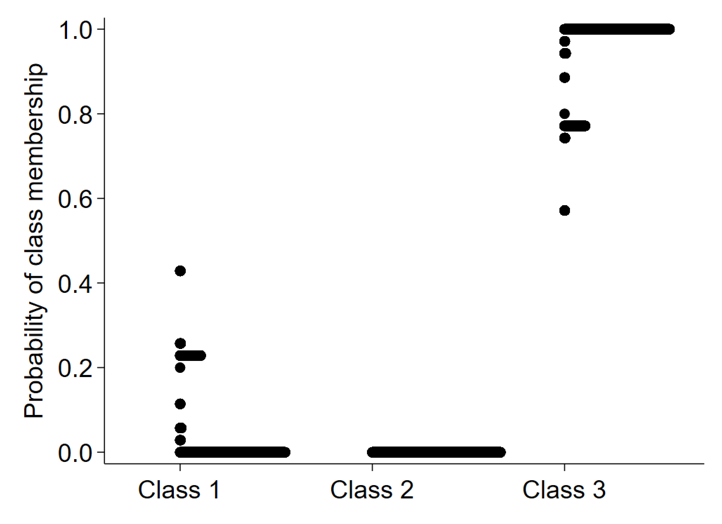 |  |

Explanation of data: models were estimated using maximum likelihood, which was calculated by summing all conditional likelihoods of each latent class multiplied by the associated latent class probabilities. The posteriori probability of a visit *i* belonging to each class *k*, *π_ik_*, was determined from this likelihood. Visits were then assigned a latent class *k* corresponding to the highest probability *π_ik_*. The figures show the distribution of probabilities for belonging to a class given the assigned class membership. Each point represents an individual consultation visit. Figure A shows the probabilities for those assigned to class 1. Figure B shows the probabilities for those assigned to class 2. Figure C shows the probabilities for those assigned to class 3. For example, the dots in the figure indicate that only few visits had a lower probability of belonging to a given class, and the longer lines (subsequent dots), indicate that the majority of visits had (almost) 100% probability of belonging to a given class, and a very low probability of belonging to another latent class.
