# Supplementary material for: Characterizing subgroups of sexual behaviors among men who have sex with men eligible for, but not using, PrEP in the Netherlands
Source: PLoS One. 2023 Apr 6;18(4):e0284056. doi: 10.1371/journal.pone.0284056 (PMC10079044; doi:10.1371/journal.pone.0284056)
Supplement: S2 Fig — Explanation of data: Bars represent the mean proportion of visits reporting each sexual behavior respectively for class 1, 2 and 3. All sexual behaviors refer to the six months prior to the visit. Number (= No.) of sexual partners refers to those with ≥6 sexual partners in the six months prior to the visit. Chemsex was defined as using cocaine, ketamine, mephedrone, gamma-hydroxybutyrate (GHB), gamma-butyrolactone (GBL), and/or crystal meth around or during sex. Bands at the top of each bar represent 95% confidence intervals, which were calculated using the delta method. (DOCX) [file pone.0284056.s006.docx]

**S2 Fig. Sexual behavior associated with increased STI risk across three latent classes, stratified by year (2019, 2020 and 2021).**

| **A. 2019** | **B. 2020** |
| --- | --- |
| 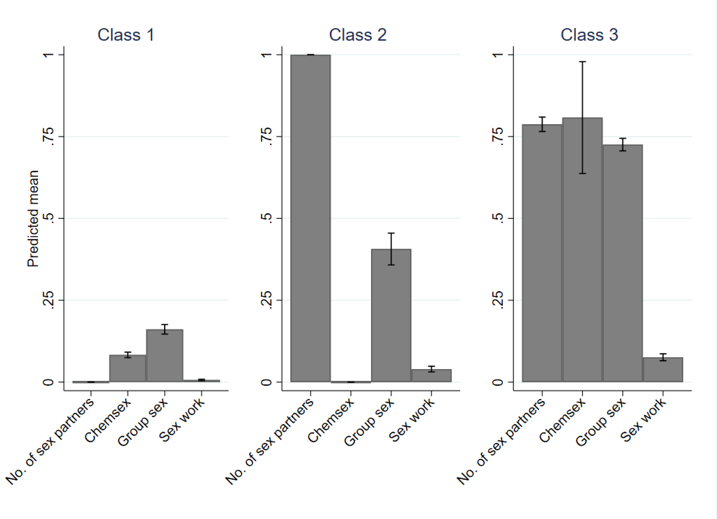 | 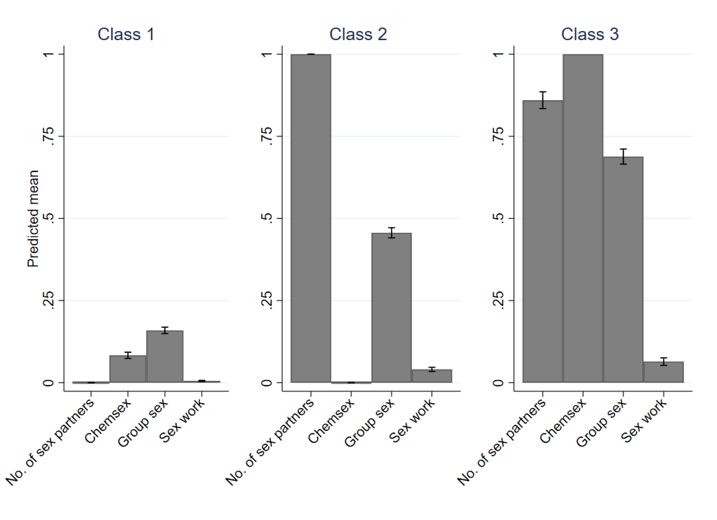 |
| **C. 2021** |  |
| 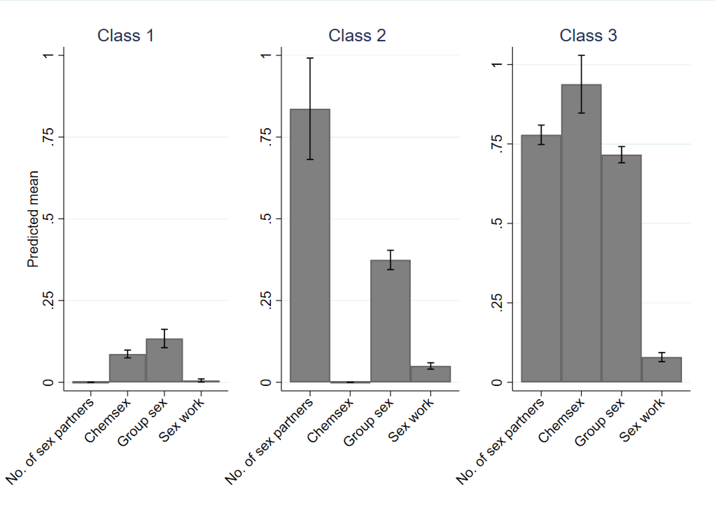 |  |

Explanation of data: Bars represent the mean proportion of visits reporting each sexual behavior respectively for class 1, 2 and 3. All sexual behaviors refer to the six months prior to the visit. Number (=No.) of sexual partners refers to those with ≥6 sexual partners in the six months prior to the visit. Chemsex was defined as using cocaine, ketamine, mephedrone, gamma-hydroxybutyrate (GHB), gamma-butyrolactone (GBL), and/or crystal meth around or during sex. Bands at the top of each bar represent 95% confidence intervals, which were calculated using the delta method.
